# Perspectives on Sotatercept in Pulmonary Arterial Hypertension

**DOI:** 10.3390/jcm13216463

**Published:** 2024-10-28

**Authors:** Rosalinda Madonna, Filippo Biondi

**Affiliations:** 1 Cardiology Division, Department of Surgical, Medical and Molecular Pathology and Critical Area, University of Pisa, 56124 Pisa, Italy; f.biondi9@studenti.unipi.it; 2 Cardiology Division, Pisa University Hospital, Via Paradisa, 2, 56124 Pisa, Italy

**Keywords:** activin, bone morphogenic protein, sotatercept, pulmonary arterial hyperension, pulmonary remodeling

## Abstract

Sotatercept acts as a type IIA-Fc activin receptor, thereby scavenging free activin from its physiological membrane receptor. Through this type of action, sotaterpect leads to a rebalancing of the proliferation and antiproliferation pathways of pulmonary smooth muscle cells in response to bone morphogenic protein (BMP). Although sotatercept has been approved as the fourth pillar of therapy for group 1 pulmonary arterial hypertension (PAH) in the United States and Europe, several studies are ongoing to broaden the application of the drug to non-Group 1 PAH. We provide an overview of the clinical and preclinical evidence of targeting the activation pathway by sotatercept in the treatment of PAH. We also discuss other potential applications of sotatercept in the context of pulmonary hypertension other than PAH group 1.

## 1. Introduction

Pulmonary arterial hypertension (PAH) is a rare and progressive disorder, primarily characterized by a remodeling of the precapillary tract of the pulmonary arterial circulation, leading to increased pulmonary vascular resistance, right heart failure, and premature death. The remodeling is due to excessive vascular cell proliferation, involving both endothelial and smooth muscle cells (SMCs) with a certain degree of venous remodeling, depending on the clinical type of PAH. Significant advances in the management of pulmonary arterial hypertension (PAH) have been achieved through the utilization of drugs that specifically target the pathways associated with endothelin (ET), prostacyclin (prostaglandin I_2_, PGI_2_) or nitric oxide (NO) [1]. Such treatments aim mainly to regulate pulmonary vascular tone by enhancing vasodilator mechanisms, rather than achieving anti-proliferative effects on pulmonary artery smooth muscle cells and endothelial cells. The inherently remodeling nature of pulmonary vasculopathy and the current absence of fully effective treatments provide a strong rationale for exploring novel therapeutic interventions that target alternative disease-related pathways.

Among the drugs of the near future, the most promising is sotatercept (brand name WINREVAIR, developed by Merck (Merck, Rahway, NJ, USA)), which has been introduced by the recent STELLAR phase III clinical trial [2]. STELLAR was a randomized, placebo-controlled study conducted on 323 patients with PAH (idiopathic, hereditary, induced by drugs or toxins, associated with connective tissue disease or associated with corrected congenital shunts), which evaluated, as a primary end point, the change from baseline in the 6 min walk distance (6MWD). Secondary end points included the time to first onset of non-fatal clinical worsening and death [2]. After 24 weeks of treatment with sotatercept, on top of background therapy, the treatment arm demonstrated improvement in the 6MWD, pulmonary vascular resistance, levels of brain natriuretic peptide prohormone N-terminal fragment, World Health Organization functional class, time to first onset of non-fatal clinical worsening and death.

Based on the STELLAR study, sotatercept received approval in the USA (March 2024) and in Europe (August 2024) for the treatment of symptomatic patients aged >18 years with PAH Group 1 (not including portopulmonary PAH, PAH associated with HIV and schistosomiasis), who are in WHO functional class (FC) II or III and stable in background PAH therapy. The addition of sotatercept to background therapy is indicated for the purpose of increasing exercise capacity, improving WHO functional class and reducing the risk of clinical worsening events. The approved drug administration begins at a dosage of 0.3 mg/kg by subcutaneous (SC) injection. If the haemoglobin levels do not increase and the platelet count does not decrease, the dosage may be increased to the recommended dose of 0.7 mg/kg once every 3 weeks.

Here, we provide an overview of the clinical and preclinical evidence pertaining to the impact of targeting the activating pathway through sotatercept on the treatment of PAH. We will also try to explore what are the actual treatment possibilities of non-Group I forms of PH by targeting this pathway.

## 2. Targeting the Activin Pathway in PAH

**Figure 1:**
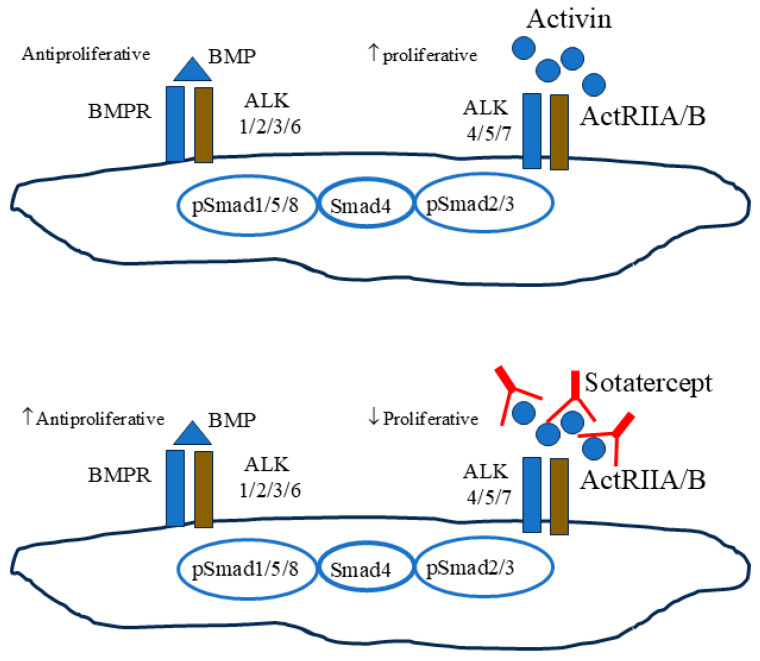
Sotatercept acts as a ligand trap for activin. Sotatercept sequesters excess ActRIIA ligands, thereby reducing ActRIIA–Smad2/3 signaling to rebalance growth-promoting and growth-inhibiting signaling. Legend to figure: BMPR, BMP receptor type 2; ALK, Activin receptor-like kinase; ActRIIA/B, activin type II receptors.

Bone morphogenetic protein (BMP), a member of the transforming growth factor-β (TGF-β) superfamily, is a key element in maintaining endothelial integrity in pulmonary arteries. BMP binds to and activates the type 2 receptor (BMPR2), which phosphorylates the transcription factor small mother against decapentaplegic (SMAD)1/5/8, allowing its translocation to the nucleus and binding to specific DNA sites. This binding inhibits DNA replication and the cell cycle in endothelial cells and smooth muscle cells. In the pulmonary circulation, BMPR2-mediated signaling thus appears as an antiproliferative mechanism, that antagonizes the proliferative arm mediated by Alk activin A-receptor system. Mutations of BMPR2 are an important factor underlying both inherited and non-hereditary forms of PAH [3,4], by reducing antiproliferative BMPR2 signaling and promoting proliferative signaling leading to pulmonary vascular remodeling. Activin A is a homodimeric polypeptide growth factor (bAbA) highly homologous to tumor growth factor (TGF)-β that interacts with the Activin A receptor like type 1 (Alk1) belonging to the TGF-beta family. Alk1 is a transmembrane protein with serine/threonine kinase activity that forms a heteromeric complex consisting of two type I receptors and two type II receptors, which phosphorylate the nuclear transcription factor SMAD 2/3/4. Upon complex formation, homodimerization of the type II receptors enhances their kinase activity. SMAD2 or SMAD3 forms a complex with SMAD4 that translocates to the nucleus and recognizes the repetitive base sequence known as the SMAD-binding element, 5′-CAGAC-3′. The discovery of activin dates back to 1986, when it was isolated for the first time in the pituitary gland as a follicle-stimulating hormone [5,6]. Subsequently, activin A has been identified in many tissues and at various stages of development, from embryogenesis to adulthood [7]. Mature activin is a 26 kDa-sized dimer formed by two carboxy-terminal domains linked by a single disulfide. The activin dimer is composed of “inhibin β subunits.” Upregulation of the activin A gene leads to several clinical conditions, such as inflammatory arthropathies, interstitial pulmonary fibrosis, inflammatory bowel disease and idiopathic or hereditary PAH. The Activin-Alk system is highly expressed in endothelial cells, where it regulates their proliferation, in vitro migration and in vivo angiogenesis. Interestingly, Alk1 co-localizes with nitric oxide synthetase (eNOS) in the endothelial caveolae, where both interact with caveolin-1 [8]. Treatment of pulmonary endothelial cells with activin A has been shown to downregulate eNOS [9]. An interaction between activin and endothelin-1 (ET-1) has also been demonstrated. ET-1 upregulates Alk expression via the Gi/RhoA/Rho kinase pathway in human pulmonary artery endothelial cells. The activation of Gi and RhoA is associated with the promoter activity of Alk via Sp-1 and the stability of Alk mRNA. Although the interaction between ET-1 and Alk has been shown only in vitro, it is highly possible that elevated ET-1 levels actually increase Alk expression in PAH patients [10]. Sotatercept [11] works by sequestering free activins, thereby restoring the balance between the activin proliferative pathway and the BMP antiproliferative pathway (Figure 1) [11]. In view of the extensive interactions between NO, ET-1 and activin, it is possible that sotatercept not only reduces the dysregulated proliferation of pulmonary circulation vessels, but also regulates their vasodilation through activin sequestration, thus removing the inhibitory brake on eNOS.

## 3. Preclinical Evidence on Targeting the Activin Signaling Pathway in PAH Models

The potential therapeutic role of targeting the activin signaling pathway via sotatercept in human PAH is supported by extensive preclinical evidence from animal models that mimic the sotatercept signaling pathway. Sotatercept is an activin receptor-type IIA-Fc (ActRIIA-Fc), an IgGq Fc fusion protein with the same extracellular domain as ACTRIIA [12]. Sugen-hypoxia rat model of severe angio-obliterative PAH showed that treatment with ActRIIa-Fc, reversed pro-inflammatory and proliferative gene expression [12]. Additionally, macrophage infiltrateon in rodent lungs was mitigated [12]. Yung et al. tested a selective transforming growth factor, beta receptor II (TGFBRII) inhibitor on monocrotaline- and hypoxia-induced pulmonary hypertension in rats and documented improvements in pulmonary vascular remodeling and hemodynamics [13]. This group reported similar results using ACTRIIA-Fc both prophylactically and after the establishment of hemodynamically severe pulmonary hypertension across various animal models [11]. Blocking ALK5 (activin-like kinase 5) proved beneficial in a rat model of pulmonary hypertension induced by monocrotaline [14]. Histologically, the favorable effects on pulmonary vascular remodeling were observed to be mediated by a decrease in early vascular cell apoptosis, adventitial cell proliferation, and metalloproteinase expression [15]. Mechanistically, inhibiting the ALK5 pathway attenuates SMAD2 phosphorylation [13]. The activin-SMAD2 pro-proliferative pathway was shown to be upregulated in the pulmonary vasculature of PAH rats [16], and this finding was recently replicated in human idiopathic PAH. Smad2 signaling was elevated in patients with idiopatic PAH (iPAH), and the concentration of activin A significantly correlated with mortality and lung transplantation [17]. In summary, a clear PAH pathogenetic model emerges: BMPR2-mediated antiproliferative signaling is reduced, while the ActRIIA-SMAD2 pathway gains prominence, leading to vascular remodeling through its pro-proliferative effects [11].

## 4. Interventional Studies and Ongoing Registered Trials on Activin Inhibitors in PAH Group 1

Activin inhibitors have been and are currently being tested in several randomized controlled trials (RCTs). The PULSAR trial [18], a phase II RCT, was a 24-week multicenter study randomizing 106 patients with PAH to subcutaneous sotatercept at two different doses (0.3 mg/kg and another dose) or a placebo. Participants had a long-standing history of PAH, with an overall time since diagnosis of 7.7 ± 5.6 years and mild to moderate functional impairment (WHO FC II-III). All patients were undergoing background PAH therapy, with up to 53% receiving triple vasodilatory therapy. The study met its primary endpoint, demonstrating a decrease in pulmonary vascular resistance (PVR) for both drug regimens and showing improvements in 6 min walking distance (6MWD) and N-terminal pro b-type natriuretic peptide (NT-pro-BNP). No serious safety issues emerged, with thrombocytopenia and increased hemoglobin levels being the main adverse events. Ninety-seven patients continued into a 24-month open-label extension, with those in the placebo arm crossing over to sotatercept. Efficacy results showed that improvements in PVR, 6MWD, and NT-pro-BNP in the crossover group were comparable to those continuing sotatercept. Adverse events were reported in 98% of participants, primarily treatment-related. Thrombocytopenia and increased hemoglobin levels remained the most common adverse effects, while telangiectasia emerged in 11% of participants. Notably, drug discontinuation occurred in almost 10% of patients, with six serious adverse events related to sotatercept, including cases of lupus erythematosus and ischemic stroke.

The STELLAR trial [2], a phase III RCT, enrolled 323 patients and randomized them toplacebo or sotatercept, starting at a dose of 0.3 mg/kg and uptitrating to a target of 0.7 mg/kg. The study met its primary endpoint, showing a statistically and clinically significant improvement in 6MWD, with an estimated difference from baseline of 40.8 m (27.5 to 54.1). Secondary endpoints were hierarchically tested, and eight were statistically significant. Importantly, the time to first occurrence of death or nonfatal clinical worsening was significantly improved in the sotatercept arm, with a hazard ratio of 0.16 (0.08 to 0.35). Time to clinical worsening is a surrogate endpoint whose relation to hospitalization and death has been shown to be more consistent than that of 6MWD [2]. Regarding drug tolerability and safety, only one patient did not reach the maximum dose, and severe adverse events were more frequent in the placebo group than in the sotatercept group. However, bleeding, thrombocytopenia, increased hemoglobin levels, and telangiectasia were more prevalent in the treatment group, consistent with data from the PULSAR trial and its extension. Further safety and efficacy data on sotatercept will be provided by the ongoing SOTERIA trial (A Long-term Follow-up Study of Sotatercept for PAH Treatment, NCT04796337), the open-label extension of STELLAR.

Moreover, because protein-based drugs can trigger immune responses that may alter their pharmacokinetics and pharmacodynamics, a parallel study was conducted on the STELLAR cohort to investigate the occurrence of anti-sotatercept neutralizing antibodies [19]. Their prevalence was notable at 6.7%, but no clear impact on treatment safety and efficacy emerged. Lastly, the SPECTRA trial [20], a phase II open-label exploratory study, assessed the impact of sotatercept on cardiac function and structure at cardiopulmonary exercise testing and cardiac magnetic resonance in a single-arm design. It concluded that treatment improved peak oxygen uptake and determined right ventricular reverse remodeling. However, the small sample size (21 participants), the pre–post study design, and the lack of a comparator are significant limitations.

Several ongoing registered clinical trials are investigating the safety and efficacy of sotatercept in specific patient populations not included in previous RCTs. MOONBEAM (NCT05597712) will clarify the safety and tolerability of activin inhibition in children with PAH. HYPERION (Study of Sotatercept in Newly Diagnosed Intermediate- and High-Risk PAH Participants, NCT04811092) is enrolling newly diagnosed PAH patients at intermediate or high risk of progression, as indicated by a REVEAL score ≥ 6 or a COMPERA score ≥ 2. ZENITH (A Study of Sotatercept in Participants With PAH WHO FC III or FC IV at High Risk of Mortality, NCT04896008) is testing sotatercept in a PAH cohort with moderate to severe functional impairment (FC III-IV) and a high risk of death (COMPERA ≥ 9).

## 5. Gaps in Evidence and Issues with Existing RCTs

Despite the impressive results of PULSAR and STELLAR, which have led to its approval as the first non-vasodilatory therapy for PAH [2], evidence on sotatercept remains limited in many respects, and several issues need to be clarified further. In particular the external validity of PULSAR and STELLAR is constrained by the stringent inclusion criteria and by the demographic and clinical characteristics of the included cohort and some aspects of the adverse effects of sotatercept need to be clarified further.

Regarding inclusion criteria both trials excluded newly diagnosed patients, those with severe functional impairment (i.e., FC WHO IV), and, perhaps most importantly, patients with cardiac and/or pulmonary comorbidities. Furthermore, STELLAR applied rigorous hemodynamic inclusion criteria to select PAH Group 1 with a pure precapillary component (precapillary wedge pressure < 15 mmHg) and advanced pulmonary vascular remodeling [pulmonary vascular resistance (PVR) > 5 wood units (WU)]. It should be noted that, while the former parameter adheres to the definition of precapillary PAH, the latter is significantly higher than the current threshold for PAH Group 1 (PVR = 2 WU). Cardiac and pulmonary comorbidities have long been known to affect the response to PAH vasodilatory therapy, specifically undermining the efficacy and/or tolerability of combined pulmonary vasodilators [21]. Given the high and rising prevalence of comorbid PAH, the current lack of data regarding sotatercept in this context could greatly limit the impact this drug will have on the PAH population as a whole. While ongoing trials will specifically try to fill the gap regarding new PAH diagnoses (NCT04811092) and PAH patients with advanced disease and/or functional limitations (NCT04896008), no registered interventional or observational study is addressing PAH with comorbidities. As has been the case with pulmonary vasodilators [22], as sotatercept enters the clinical practice, useful data about its efficacy and safety in the real-world highly comorbid setting will come from registries. Yet, this type of information is prone to bias [23,24] and certainly cannot make up for the exclusion of comorbid PAH from randomized controlled trials. Hence RCTs dedicated to the comorbid PAH phenotype are sorely needed.

Concerning the demographic and clinical characteristics of the study cohort, Black and Asian patients were barely represented despite reports that ethnicity has an influence over prognosis [25] and response to PAH therapies [26]. Future observational studies and RCTs will have to address the issue and enroll a sufficient number of patients for each ethnical group so as to perform subgroup analyses and clarify the impact of ethnicity on sotatercept efficacy and safety. Moreover, cohorts in PULSAR and STELLAR were both affected by long-standing PAH (mean time since diagnosis of 8.8 + −7.0 years and 7.7 + −5.6) and were atypical for the high proportion of subjects receiving double or triple combination therapy (94.5% in STELLAR vs. 46.3 % in the COMPERA registry). Considering that the survival probability of PAH patients shows a steeper decline in the first years since diagnosis and tends to stabilize at a later stage CIT, RCTs on sotatercept may have enrolled a cohort with a high proportion of optimally-treated long survivors. Moreover, this cohort of long survivors seems atypical because of the high proportion of triple therapy, which current guidelines for high-risk patients indicate a rapid decline in survival [27]. This may raise the question of whether and to what degree the benefits of sotatercept may apply to more typical, newly diagnosed PAH patients. The HYPERION trial (NCT04811092), a phase III multicenter RCT, will address this specific gap by enrolling patients diagnosed with PAH within 12 months, but its completion is expected in 5 years’ time. In the meantime, a subgroup analysis of the STELLAR cohort according to different time spans since diagnosis could have yielded some valuable information.

Regarding the hemodynamic effect of sotatercept, it has been shown to exhibit a peculiar pattern that differs from that of pulmonary vasodilators. In particular, a dedicated analysis of the STELLAR trial [2] found that sotatercept reduced PVR not through an increase in cardiac output, which remained stable, but through a decrease in mean pulmonary arterial pressure (mPAP). On one hand, this aligns with the notion that sotatercept has a systemic vasoconstrictive rather than vasodilatory effect [28]; on the other hand, it challenges the concept that cardiac output, not mPAP, correlates with survival in PAH [29]. Moreover, as noted by Rubin and Naeije [29], it is curious that sotatercept’s favorable effect on hemodynamics seems to manifest in a relatively limited time frame and does not increase over time [30]. The synergism between sotatercept and other vasodilatory drugs in background therapy may explain the early effect of sotatercept, also considering the extensive interaction that sotatercept has with the NO and ET-1 pathways (see chapter 2). Moreover, while reverse remodeling has been widely associated with pulmonary vasodilator therapies across the diverse types of PAH [31], this effect has not been described in sotatercept-treated patients yet. Further information on the interplay between sotatercept and different PAH drug classes could have been inferred by performing a subgroup analysis of the STELLAR cohort according to not only to the number of drugs but also to the type of drugs. Specific combinations may indeed be more effective than others. It has to be noted, however, that the STELLAR trial was likely underpowered to detect the possible benefit of choosing one drug combination over another. Ongoing RCTs and future metanalyses will hopefully investigate this possibility.

Regarding adverse effects, sotatercept has a well-characterized erythropoiesis-stimulating action and has been tested for treating anemia in various conditions [32,33]. As expected, sotatercept was associated with a modest increase in hemoglobin (Hb) in RCTs enrolling patients with PAH, with the mean Hb rising by 1.3 mg/dL [2]. Of note, Both PAH and heart failure are associated with absolute and functional iron deficiency [34], and anemia has been widely linked to worse outcomes in both conditions [35,36]. However, erythropoietin seems to be elevated in heart failure (HF) and to positively correlate with mortality. Moreover, darpoietin in HF has failed to show any beneficial effect and was associated with a higher risk of thromboembolic events [37]. Erythropoietin has also been found to be elevated in PAH [38] and has been recently shown to exert an apparently favorable effect on the pulmonary vasculature by promoting homing and endothelial cell differentiation in rat bone marrow stem cells [39]. Whether the stimulation of erythropoiesis is an adverse effect or a mediator of sotatercept efficacy is an intriguing question that cannot yet be answered. Still, useful information could arise from a mediation analysis using data from the main RCTs. Moreover, given the well-known link [37,40] between erythropoietin and erythropoietin stimulating agents and thrombotic events, a close monitoring of the incidence of CV adverse events in sotatercept-treated individuals is certainly warranted. It has to be noted, however, that no indication of a heightened risk of thrombosis and thromboembolism has emerged from PULSAR and STELLAR, with only one patient affected by stroke in the treatment arm of the former. Yet, because sotatercept has a long term use, an increase in the thrombotic risk may become evident in the future and will need to be promptly recognized. Another issue with RCTs on sotatercept is the choice of 6MWD as the primary endpoint of the STELLAR trial, which marks a step back from the more recent RCTs in the PAH space. Registration studies of PAH drugs have used the performance at the 6MWD test as the primary outcome up to the 2013 FREEDOM-C2 study [41]. From there on, morbidity and mortality composite primary endpoints were opted for. In particular, the concept of time to clinical worsening (TTCW) was first successfully used in a study on macitentan [42] and was later confirmed as a valid primary endpoint for registration trials. Indeed, TTCW was shown to have a relation to hospitalization and death, which is more consistent than that of the 6MWD [43] and should be the primary endpoint of future RCTs testing sotatercept. Of note, STELLAR did show an improvement in TTCW, which was included in the secondary endpoints. This, however, does not necessarily translate into a decrease in mortality [44], which will have to be assessed in longer-term studies and/or registries.

## 6. Could the Benefits of Sotatercept Therapy Apply to Non-Group 1 PH?

PAH Group 1 represents only a small fraction of PH cases, which are mostly associated with left heart disease (PH Group 2) [45]. HF-associated PH lacks effective dedicated therapies, and its management is limited to optimal treatment of the underlying left heart disease [46]. Great efforts have been made to test pulmonary vasodilators in this setting, but dedicated RCTs have yielded, at best, mixed results [47], and no PAH-specific drug class is formally recommended in PH Group 2. Interestingly, a link between the TGF signaling pathway and HF (both acute and chronic) has been suggested by a number of observational studies, to the point that it has been proposed as an additional pillar in the maladaptive mechanisms fueling HF [48]. Activin A was shown to negatively correlate with left ventricle ejection fraction (LVEF) during hospitalization and with worse survival at six months. These clinical findings are supported by a wealth of preclinical studies. Activin A can stimulate myocardial cell apoptosis [49] through endoplasmic reticulum stress. Consistently, inhibition of the ACTRII receptor in aged mice and mice with non-ischemic HF resulted in improved cardiac function [50]. Tan et al. [51] used an ALK5 inhibitor in a rat model of HF induced by myocardial infarction. Treatment was associated with an improvement in systolic heart dysfunction. Authors hypothesized that TGF-beta inhibition may reduce fibrosis if administered at the right time frame—neither too early nor too late after the inciting event. A key study by Roh et al. [48] leveraged multiple human cohorts to prove that circulating activins increase in human aging and HF. Concurrently, ACTRII signaling was also increased in established animal models of aging and HF. These observational findings are confirmed by an intervention trial in which a sotatercept analog was shown to revert remodeling of the pulmonary vasculature in rats with HF complicated by combined pre- and post-PH, a finding not replicated with sildenafil. Whether these preclinical data can translate to clinical value will be clarified by the ongoing CADENCE trial (NCT04945460), a phase II, double-blind, randomized, placebo-controlled study evaluating the efficacy and safety of sotatercept in combined pre- and post-PH-associated with HF with preserved EF (HFpEF). PH-HFpEF in the combined pre and post capillary from (Cpc) represents the form of PH Group 2 that most closely resembles PAH, primarily due to the underlying vascular remodeling. Data from registries suggest that CpcPH associated to HFpEF can be safely treated with PDE5i, so that current guidelines do not issue a formal contraindication to their use in this setting CIT. This fact, together with the common pathological background, suggests that PH-HFpEF may have the highest likelihood of responding positively to sotatercept, making it a crucial area for further exploration and treatment strategies. Even specific forms of PAH group 1, such as portopulmonary hypertension (which has a pathogenetic root very different from the other forms of this group), can be treated with ERA or with PDEi without incurring the risk of haemodynamic worsening following the induction of hepatorenal syndrome [31]. The association of a vasodilator such as PDE5i and an antiremodelling agent such as sotatercept is not investigated in ongoing trials.

Activin and the TGF pathway have also been linked to lung disease and COPD in particular. A study by Verhamme et al. has suggested that a dysregulation of the activin pathway plays a role in a mice model of smoke-induced obstructive lung disease [52], while Zhou et al. have linked activin A to muscle wasting in COPD [53]. Moroever, the activin inhibitor bimagrumab was shown to safely increase skeletal muscle mass in COPD patients [54]. Interestingly, skeletal muscle atrophy and dysfunction has been widely described in PAH [55] and could be a yet unrecognized target of sotatercept, as already suggested in this paper. Group III PH is a possible and frequent complication of lung disease [56] with very limited therapeutical options. RCTs have been conducted on different drug classes in group III PH, but the only promising results came from inhaled Treprostinil [57], currently a class IIB recommendation (PMID: 36017548). To the best of our knowledge, the possible role of sotatercept in this condition has never been investigated despite evidence that the TGF pathway could be directly implied in PH associated with lung disease [58]. Future studies are warranted to clarify whether sotatercept may be a safe and effective option for group III PH, either alone or in combination to endothelin receptor antagonists.

## 7. Conclusions and Future Perspectives

Sotatercept is the fourth pillar in the armamentarium against PAH. The drug further improves exercise capacity, hemodynamics symptomatology, quality-of-life metrics, and time to clinical worsening. Sotatercept’s favorable effect on hemodynamics and survival manifest in a relatively limited time frame, an effect likely due to the synergism between sotatercept and other vasodilatory drugs in background therapy. Understanding the molecular-level interactions of sotatercept with existing PAH therapies could help optimize its use in combination therapies. With regard to the timing of the start of therapy, according to the current state of knowledge, sotatercept is certainly not indicated as a monotherapy or as initial combination therapy but rather as a sequential combination therapy in stable patients in FC-WHO functional class II/III. HYPERION and ZENITH will clarify the possible indication of the drug in high-risk patients in FC-WHO functional class III and IV, while the SOTERIA study will shed light on the long-term efficacy and safety.

However, it has a non-negligible effect on erythropoiesis and the safety and efficacy of the drug in the presence of co-morbidities are not known. Several clinical trials are underway to evaluate its efficacy in other PH groups such as Group 2. There is still no evaluation of the safety and efficacy of the drug in pulmonary vein obstructive disease (PVOD) or porto-pulmonary arterial hypertension. Therefore, further studies are needed to better define the safety of the drug and its efficacy, especially in the long term.

## Data Availability

No new data were created or analyzed in this study. Data sharing is not applicable to this article.
